# Effect and Mechanism of Pharmaceutical Excipients on Berberine to Alleviate Ulcerative Colitis via Regulating Gut Microbiota

**DOI:** 10.3390/molecules27185997

**Published:** 2022-09-15

**Authors:** Chenyang Wu, Tingting Zheng, Huan Chen, Peizhi Zou, Mengxue Zhang, Jinrui Wang, Nan Li, Yun Zhang, Ying Li, Zhengqi Dong

**Affiliations:** 1 Drug Delivery Research Center, Institute of Medicinal Plant Development, Chinese Academy of Medical Sciences & Peking Union Medical College, Beijing 100193, China; 2 Department of Pharmacy, Faculty of Pharmacy, Hebei Medical University, Shijiazhuang 050017, China; 3 Faculty of Pharmacy, Heilongjiang University of Chinese Medicine, Harbin 150040, China; 4 Key Laboratory of Bioactive Substances and Resources Utilization of Chinese Herbal Medicine, Ministry of Education, Institute of Medicinal Plant Development, Chinese Academy of Medical Sciences & Peking Union Medical College, Beijing 100193, China; 5 Key Laboratory of New Drug Discovery Based on Classic Chinese Medicine Prescription, Chinese Academy of Medical Sciences, Beijing 100193, China; 6 Beijing Key Laboratory of Innovative Drug Discovery of Traditional Chinese Medicine (Natural Medicine) and Translational Medicine, Institute of Medicinal Plant Development, Chinese Academy of Medical Sciences & Peking Union Medical College, Beijing 100193, China

**Keywords:** ulcerative colitis, berberine, pharmaceutical excipients, intestinal barrier, gut microbiota

## Abstract

Background: Various potential effect of drugs on alleviating diseases by regulating intestinal microbiome as well as the pharmaceutical excipients on gut microbiota has been revealed. However, the interaction between them is rarely investigated. Methods: Histological analysis, immunohistochemistry analysis, enzyme-linked immunosorbent assay (ELISA) analysis, RT-qPCR, and 16S rRNA analysis were utilized to explore the effect mechanism of the five excipients including hydroxypropyl methylcellulose (HPMC) F4M, Eudragit (EU) S100, chitosan (CT), pectin (PT), and rheum officinale polysaccharide (DHP) on berberine (BBR) to cure UC. Results: The combined BBR with PT and DHP group exhibited better therapeutic efficacy of UC with significantly increased colon length, and decreased hematoxylin-eosin (H&E) scores than other groups. Furthermore, the expression of tight junction ZO-1 and occludin in colon tissue were upregulated, and claudin-2 was downregulated. Ultimately, the serum content of tumor necrosis (TNF)-α, interleukin (IL)-1β, and IL-6 was decreased. Moreover, the combined BBR with PT significantly promoted the restoration of gut microbiota. The relative abundance of Firmicutes and Lactobacillus was significantly increased by the supplement of PT and DHP, and the relative abundance of Proteobacteria was downregulated. Conclusions: Our study may provide a new perspective that the selection of pharmaceutical excipients could be a crucial factor affecting the drugs’ therapeutic efficiency outcome.

## 1. Introduction

Inflammatory bowel disease (IBD) has emerged as a growing problem in the world, and the prevalence of IBD in the Western world was up to 0.5% of the general population in 2015 [1]. As the most crucial part of IBD, ulcerative colitis (UC) has the characteristics of recurrent diseases accompanied by continuous diarrhea, abdominal pain, and other clinical symptoms [2,3,4,5], which greatly reduced the life quality of patients. 

Berberine (BBR), is a natural extract predominantly derived from *Coptis chinensis* and *Berberis vulgaris* with an amino structure. At present, numerous researchers have confirmed that BBR has excellent antibacterial, hypoglycemic, and lipid-lowering properties and efficacy in the treatment of UC and nervous system diseases including Alzheimer’s and Parkinson’s disease [6,7]. Increasing evidence has suggested that BBR could ameliorate UC by improving metabolic disorders [8], decreasing the expression of oncostatin M (OSM) and oncostatin M receptor (OSMR), and inhibiting the overactivation of human intestinal stromal cells through the OSM-mediated Janus kinase (JAK) signal transducer and activator of transcription (STAT) pathway [9], modifying gut microbiota and regulating the balance of Regulatory T cell (Treg)/ T helper cell 17 (Th17) [10]. It is worth noting that BBR with a variety of therapeutic effects, has extremely low bioactivity [11], and more attention has been paid to relative research to explain the contradictory phenomenon. However, the traditional concept of drug bioavailability has been updated by scholars with new meanings. From the viewpoint of the gut microbiota, there are four ways to affect the bioavailability of drugs, including an increase of beneficial metabolites from active substances by enriching specific gut bacteria [12], a decrease in detrimental metabolites from active compounds by regulating the gut microbiota and inhibition of transforming active substance into inactive forms via controlling specific gut microbiota.

The research on the influence of the intestine flora in the occurrence and development of UC has been intensified [13], and the regulating of balance in gut microbiota, diet control, anti-oxidation, and immune regulation are all regarded as potential treatments to alleviate UC. Merely from the perspective of gut microbiota, a stable flora environment can repair the intestinal barrier function, which reverses the trend of increased intestinal permeability caused by diseases. Some intestinal-derived substances can induce overall inflammation of our body, such as lipopolysaccharide (LPS), its possibility of entering the body’s circulation is significantly reduced, and the overall inflammation level is effectively controlled. In addition, the flora metabolites also affect the level of our health, and the production of beneficial metabolic molecules such as short-chain fatty acids (SCFAs) [14], will speed up the recovery of the body to a much healthier state [15]. On the contrary, harmful metabolic have the opposite effect. From the perspective of the interaction between the intestine and other organs, a drug intervention may produce part of the effect of curing diseases through the intestine–other organ axis [16].

Much work has so far focused on the relationship between food or drug additives and intestinal flora [17], playing a vital role in our dietary health and medication safety. Generally speaking, this effect can be summarized into three major trends. Firstly, pharmaceutical excipients cause certain damage to body tissues by enriching pathogenic bacteria or disrupting the overall balance of the flora, gut dysbiosis may be generated by TiO_2_ NP-containing foods [18]. Second, pharmaceutical excipients exhibit a specific influence on the composition of gut microbiota without any damage to the body’s health [19]. Finally, the bioavailability of the drug may be increased or decreased by pharmaceutical excipients in the way of regulating the specific gut microbiota [20], meantime, the excipient itself can have a beneficial effect on the body by regulating intestinal flora [21].

**Figure 1:**
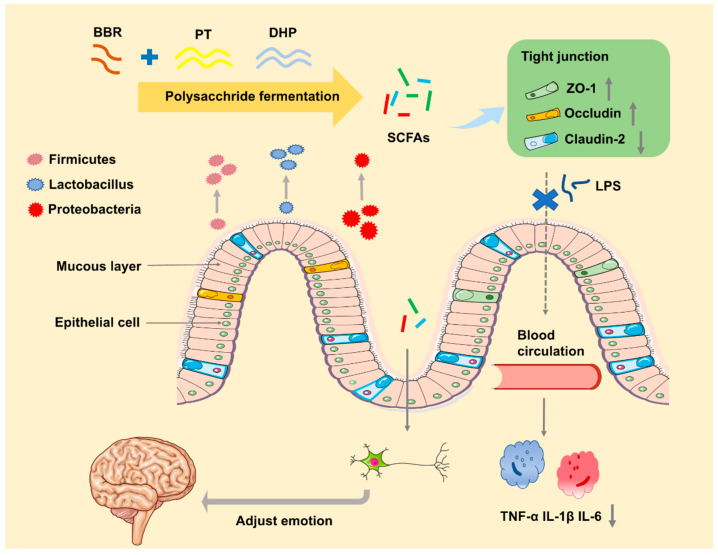
The mechanism of PT and DHP plays a vital role in alleviating the symptom of colitis.

Based on the object of investigating the interaction mechanism of excipients and BBR to cure UC via gut microbiota, five commonly used excipients: hydroxypropyl methylcellulose (HPMC) F4M, Eudragit (EU) S100, chitosan (CT), pectin (PT) and *rheum officinale* polysaccharide (DHP) for the colon-targeted delivery system were selected to explore their effect on the treatment of dextran sulphate sodium (DSS)-induced colitis mice. The mechanism (Figure 1) was investigated by analyzing the gut microbiota, gut integrity, and immunity. Further, the discrepancy between all groups will be analyzed to explore the potential effect of pharmaceutical excipients on BBR to cure UC.

## 2. Results

### 2.1. Effect of PT and DHP Supplement on Colonic Damage by DSS

**Figure 2:**
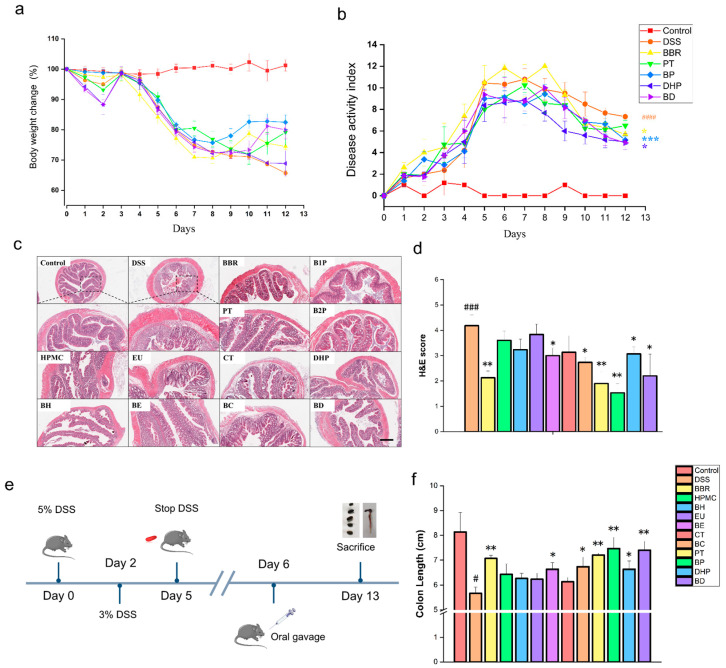
(**a**) Daily body weight change. (**b**) DAI index was calculated according to the comprehensive examination of body weight change, stool consistency, and occult blood scores. (**c**) H&E staining images of every group. (**d**) H&E scores were analyzed by the image of H&E staining. (**e**) Experimental design. (**f**) Colon lengths were recorded. (Significance was assessed by using *t*-test, ns, not significant; # *p* < 0.05, ### *p* < 0.001, #### *p* < 0.0001, compared with the control group; * *p* < 0.05, ** *p* < 0.01, *** *p* < 0.001, compared with the DSS group).

As one of the most classical IBD animal models, DSS-induced colitis mice was used for the exploration of potential influence in groups accompanied by different treatment. After receiving 5% DSS solution (in drinking water), all mice began losing weight and this extended to day 3; on that day, the concentration of DSS solution was turned down to 3% (in drinking water) until day five (Figure 2e). The characterization of colitis including weight change, fecal consistency, and bloody diarrhea was observed once a day to evaluate the establishment of the colitis model (Figure 2a,b). We found that the feces of mice given DSS solution got moist and sticky from day 3 to 6 and presented transparent blood on day 4, indicating that there was a significant discrepancy in disease activity index (DAI) between the groups with DSS intervention and the normal control. Groups of mice receiving PT and DHP interventions showed a higher probability of survival compared to the groups in the presence of HPMC, EU, and CT interventions. The combined administration group of BBR and PT reversed the symptoms of body weight loss in mice with colitis to the greatest extent (Figure 2a). After 12 days of molding, the highest DAI was observed in the DSS group (Figure 2b). Compared to the DSS group, the BBR, BD, and BP groups all significantly reduced the DAI of the mice (Figure 2b). The histopathological changes in the colon tissue were observed by H&E staining. In the control group, the colonic histological structure remained intact and undamaged, with neatly arranged cupulae, crypt, and intestinal glands (Figure 2c), and lower hematoxylin-eosin (H&E) scores. Compared with the control group, the DSS intervention induced damage to the colonic epithelial barrier, with erosion and severe inflammatory infiltration. A significantly higher H&E score was observed in the DSS group than in the control group. Compared with the physically mixed group, the colonic tissue damage was still more severe in the group only with the intervention of excipients, with the PT group and the DHP group being slightly less serious. The trend of the physical mixed group remained the same as that, and the BE, BC, and BP groups all showed some degree of improvement, with the best effect in the BP group (Figure 2c). The H&E scores of all groups indicated that the treatment of PT, as well as combined BBR with PT, protected the colon tissues of DSS-induced mice from destruction, and the beneficial function of the DHP group and the combined administration group of DHP and BBR were second only to it (Figure 2d). The average length of the colon in the control group was about 8 cm, and it was shortened to less than 6 cm in the DSS group. The intervention of BBR significantly inhibited the colon shortening caused by DSS and maintained it at about 7 cm, while in the excipients group, only the PT and DHP group significantly inhibited colon shortening. The PT group was basically the same as that in the BBR group, the DHP group was slightly inferior to the PT group. Moreover, in the physically mixed group, the BE, BC, BP, and BD groups all had a certain degree of improvement. The effect of the BP and BD group in protecting colon tissue is more prominent (Figure 2f).

### 2.2. Effect of PT and DHP Supplement on the Expression of Tight Junction

**Figure 3:**
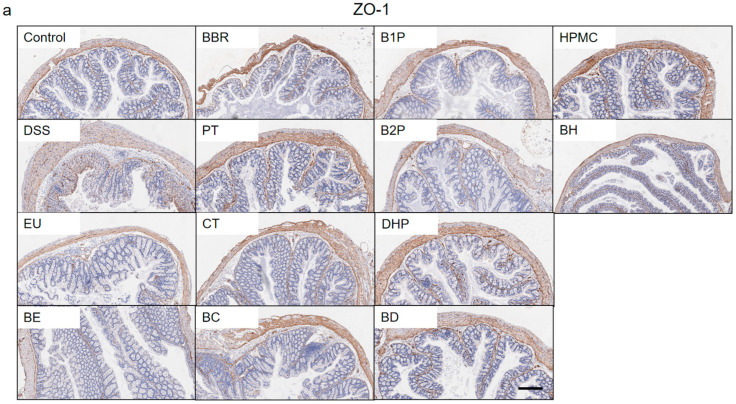

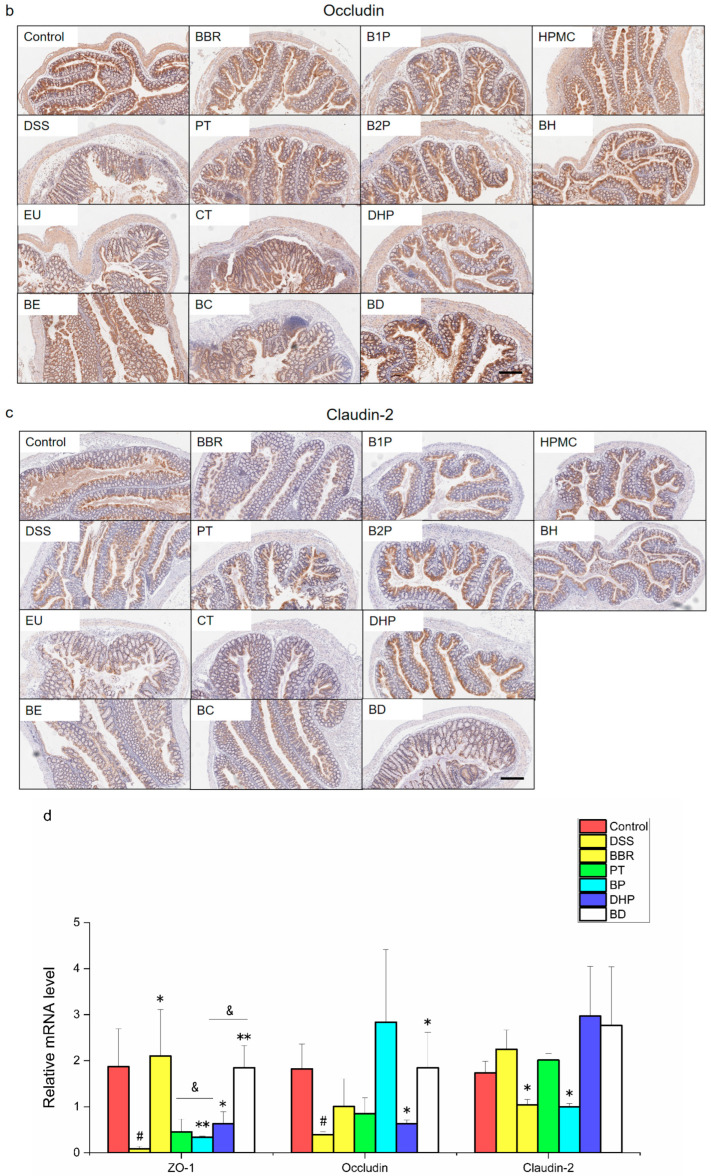
(**a**–**c**) Immunohistochemical staining of ZO-1, occludin, and claudin-2. (**d**) Colonic mRNA levels of ZO-1, occludin, and claudin-2. (Significance was assessed by using *t*-test, ns, not significant; # *p* < 0.05, compared with the control group; * *p* < 0.05, ** *p* < 0.01, compared with the DSS group; & *p* < 0.05, compared with the group combined with BBR).

A large number of studies demonstrated that the expression of intestinal tight junction protein was closely related to the occurrence and development of colitis. The inflammatory activity was positively correlated with the expression of claudin-1 and claudin-2, contrary to the expression trend of zonula occludens-1 (ZO-1) and occludin. Then we conducted further experiments in order to explore and clarify if or how to repair the intestine barrier by PT and DHP, which may facilitate the alleviation of colitis. As the Immunohistochemistry (IHC) results (Figure 3a–c) showed that the protein expressions of ZO-1 and occludin in the colon tissue of the DSS group were significantly downregulated, which indicated the intestinal barrier function was destroyed. However, treatment using BBR facilitated repairing the intestine barrier through upregulating the protein expression of ZO-1 and occludin. At the same time, the intervention of PT and DHP effectively increased the expressions of these two tight junction proteins, which was more pronounced in the BP and BD group. It should be pointed out that the protein expression of ZO-1 was affected mainly by the intervention of PT; however, DHP played a vital role in the expression of occludin. Contrary to ZO-1 and occludin, the protein expression of claudin-2 was upregulated in the DSS group, which was remarkably reversed in the BD group. All the results showed that the intervention of PT and DHP produced a positive effect on repairing the damaged intestine barrier. To gain more insight, we detected the colonic mRNA expression, including ZO-1, occludin, and claudin-2, then we found that the expression of ZO-1 and occludin was upregulated; in the meantime, the expression of claudin-2 was downregulated, which was consistent with the observation from IHC. In conclusion, the results from IHC and Q-PCR (Figure 3d) suggested that PT and DHP had a potential function in regulating the expression of intestine tight junction protein, and combined BBR with these two excipients did best to body health and optimally promoted the relief of colitis.

### 2.3. Effect of PT and DHP Supplement on the Serum Inflammation

**Figure 4:**
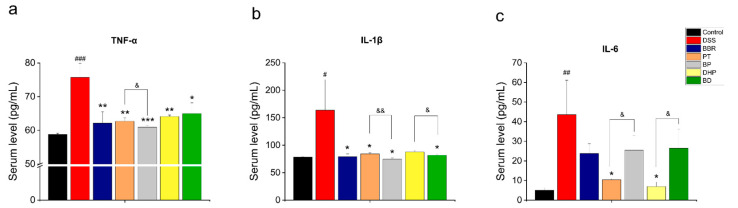
(**a**) The serum concentration of TNF-α; (**b**) the serum concentration of IL-1β; (**c**) the concentration of IL-6. Significance between every two groups was calculated by using *t*-test. # *p* < 0.05, ## *p* < 0.01, ### *p* < 0.001, compared with the control group; * *p* < 0.05, ** *p* < 0.01, *** *p* < 0.001, compared with the DSS group; & *p* < 0.05, && *p* < 0.01, compared with the combination group.

The level of intestinal inflammation of mice was evaluated by detecting the expression of serum cytokines, As expected, the therapeutic effect of BBR corresponds with previous research, demonstrating excellent anti-inflammatory results by significantly downregulating the level of TNF-α and IL-1β (Figure 4a,b), the concentration of IL-6 was decreased without significant change (Figure 4c). Judging from the results, combined BBR with PT showed superior anti-inflammation ability than the PT, DHP and BD group in reducing the amount of TNF-α and IL-1β. However, the single administration group of PT and DHP reduced the plasma level of IL-6 to a greater extent than the BP and BD group with a significant difference.

### 2.4. Effect of PT and DHP Supplement on the Microbial Imbalance

**Figure 5:**
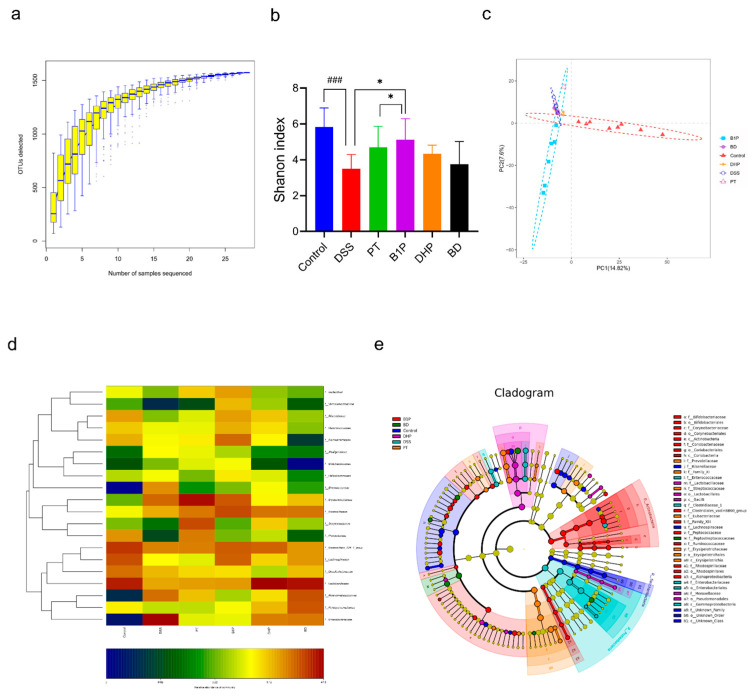

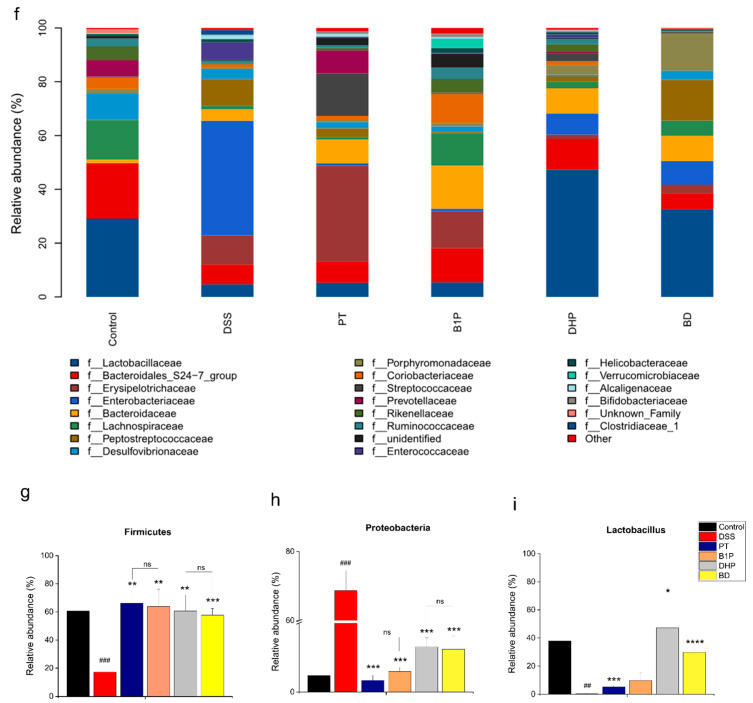
16S rRNA gene sequencing analysis of gut microbiota regulated by PT, DHP, the combination of BBR with PT, and DHP. (**a**) Rarefaction curves. (**b**) Shannon index showed the α–diversity of the microbial community. (**c**) PLS–DA showed the β–diversity of gut microbiota. (**d**) Heatmap showed the changes in relative abundance of microbial compositional profiling at the genus level. (**e**) Cladogram exhibited the difference in richness and the group with a significant difference in abundance. (**f**) Community histogram showed the compositional discrepancy between various groups at family level. (**g**–**i**) Relative abundance of Firmicutes, Proteobacteria, and Lactobacillus. The *t*–test was utilized for statistical analysis. ns, not significant, ## *p* < 0.01, ### *p* < 0.001, compared with the control group; * *p* < 0.05, ** *p* < 0.01, *** *p* < 0.001, **** *p* < 0.0001, compared with the DSS group.

It is reported that the relationship between gut microbiota and the IBD is mainly reflected in three major aspects, including the infection of an enteric pathogen, the excessive level of translocation of intestinal bacteria across the intestinal barrier, and proportionally imbalanced “beneficial” and “detrimental” bacteria [22]. To identify if and how PT and DHP regulate the gut microbiota and determine the potential influence in the function of affecting special microbiome by BBR in DSS-induced colitis mice, we performed 16S rRNA gene sequencing of feces collected from mice. Rarefaction curves (Figure 5a) showed that bacterial species richness is positively related to the sampling depth, indicating the sequencing effort was sufficient. Compared with normal controls, the DSS-induced colitis mice were characterized by a significantly decreased Shannon index, indicating the disturbance of the gut microbiome in the DSS-induced mice, which is consistent with previous research results [23]. PT and DHP alleviated the imbalance condition of the gut community by upregulating the abundance (α-diversity) of microbiota, but we found no significant distinction between the three groups (the DSS group, the PT group, and the DHP group); however, combined BBR with PT significantly promoted the restoration of gut microbiota compared with the DSS group (Figure 5b), which indicated that the optimal therapeutic effect was generated by the combination of BBR and PT. Then, partial least squares discriminant analysis (Figure 5c) was utilized for Beta analysis of compositional features in various groups, and the transparent segregation of the overall gut community after drug intervention was observed. Among them, the combined administration group of BBR and PT has the most obvious changes. Cladograms (Figure 5e) were used to identify the taxa that were significantly changed among all groups according to the relative abundance. The general composition of each group at the family level was exhibited in the heatmap (Figure 5d) and bar chart (Figure 5f), respectively, indicating that there were significant differences in the microbial composition of each group at different levels. According to the OTU classification, the specific composition of each group at various taxonomic levels was compared and evaluated. The abundance of Proteobacteria that was highly related to UC increased differently in the DSS group compared with the blank control due to the inducement of DSS (Figure 5h) [24], which was possibly brought about by the disorders of the intestinal microenvironment. The relative counts of Proteobacteria were significantly downregulated by the combined treatment of PT and BBR, as well as DHP and BBR. Interestingly, the same trend was produced in the PT group and DHP group, there was no significant change between the separate and combined treatment group. Contrary to the change of Proteobacteria, the relative abundance of Firmicutes was upregulated by PT and DHP (Figure 5g), which was found with the potential function of plant polysaccharide fermentation [25]. It is suggested in a previous study that Lactobacillus might actually protect the mucosa from inappropriate inflammatory responses that could damage the host [13], and this was also reflected in our research results. The relative abundance of Lactobacillus was significantly upregulated in the PT, DHP, and BD groups compared to the DSS group, indicating the superiority of plant polysaccharide intervention (Figure 5i). In summary, we found that the combined intervention of BBR with PT and DHP efficiently regulated the gut microbiota composition in the DSS-induced UC model mice. Firmicutes, Proteobacteria, and Proteobacteria.

### 2.5. Screen of the Ratio of BBR to PT via Evaluating the Abundance of the Gut Community 

**Figure 6:**
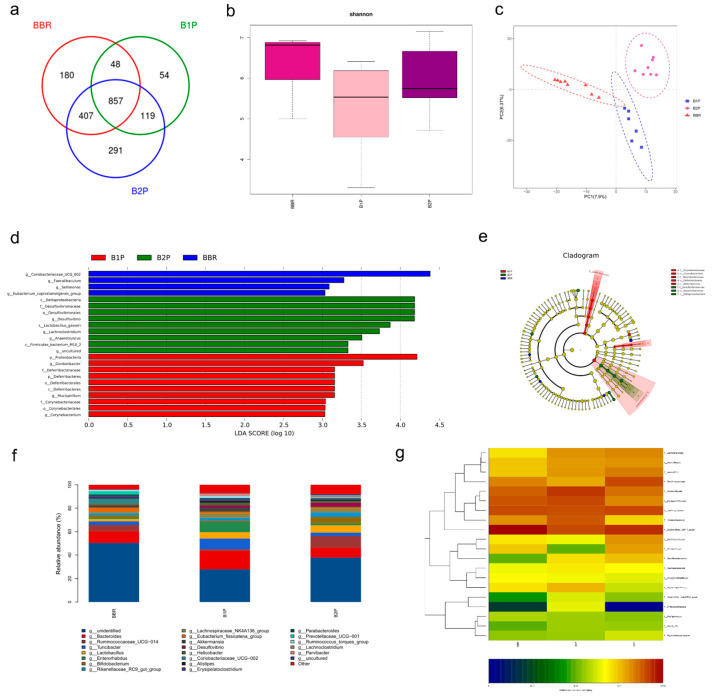
16S rRNA gene sequencing analysis of gut microbiota regulated by combined BBR and PT. (**a**) Venn diagram displayed the degree of overlap of bacterial OTUs. (**b**) Shannon index showed the α–diversity of the microbial community. (**c**) NMDS analysis showed the β–diversity of gut microbiota. (**d**) Linear discriminant analysis (LDA) showed the significantly abundant microbiome altered at levels from phylum to genus. (**e**) Cladogram exhibited the difference in richness and the group with a significant difference in abundance. (**f**) Community histogram showed the compositional discrepancy between various groups at the genus level. (**g**) Heatmap showed the changes in relative abundance of microbial compositional profiling at the family level.

On the basis of the above research that was implemented in our laboratory, we found that the supplement of PT exhibited excellent beneficial biological effects on BBR to facilitate the relief of colitis symptoms through improving intestinal barrier function and regulating gut microbiota in DSS-induced mice. Furthermore, the drug ratio of BBR to PT was explored according to the regulation of gut microbiota. The middle circle in the Venn diagram (Figure 6a) indicates the number of shared OTUs in these three groups was 857, and the number of OTUs that only existed in the B2P group was the largest, which was 291. The Shannon index showed that α-diversity was upregulated to a greater extent in the B2P group than in the B1P group (Figure 6b). However, there was no significant change compared with the BBR group. β–diversity analysis using NMDS analysis (Figure 6c) indicated the clear segregation of the overall gut microbiome in the BBR, B1P, and B2P groups. LDA (Figure 6d) showed that there were 22 OTUs significantly different in the three groups, only 4 OTUs were higher in the BBR group, and 9 OTUs and 10 OTUs were higher in the B2P group and B1P group, respectively. Cladograms (Figure 6e) were used to identify the taxa that were significantly changed among all groups according to the relative abundance. The general composition of each group at the genus level (Figure 6f) was exhibited in the bar chart. The relative abundance of Lactobacillus was upregulated in B1P and B2P groups compared to the BBR group. Similarly, the same trend was shown in the relative abundance of Bifidobacterium, which was obviously upregulated in the B2P group. It is indicated in the heatmap (Figure 6g) that the relative abundance of Lachnospiraceae in the B2P group was higher than in the BBR and B1P group, which was downregulated in the DSS group. This trend of downregulation was consistent with previous research [24].

## 3. Discussion

The DSS-induced mouse colitis model, as the most proven and widely applicable technology among IBD modeling methods [21], was deemed suitable for investigating the pathogenesis and therapeutic option of UC. During the process of DSS intervention, selected DSS aqueous solutions with different concentration gradients were used for acute colitis induction. The mice were treated with a 5% DSS aqueous solution for two days before the model was established, and a 3% DSS aqueous solution was used for three days after the model was established. Previous studies have shown that 5% DSS aqueous solution intervention will increase the mortality of mice, consistent with the results from our study, accompanied by a large number of deaths on the ninth and tenth days. Based on the result, we conducted an analysis and comparison of the survival rate between the mice groups, and the combined administration group of BBR and PT was regarded as the best group according to the survival curve. Although the physical difference of mice may have a specific impact on their survival, which can be eliminated by random grouping before the experiment.

In this study, five commonly used excipients were screened, including HPMC, EU, CT, PT, and DHP, all of which were used for the preparation of the formulation for UC. However, the interaction of excipients with the health of the body and the intestinal environment varies. It is known that some excipients can have a certain beneficial effect on the body [26,27,28,29,30] or exert a better effect by combining with other drugs [31,32]. It was pointed out that PT supplement significantly strengthened the anti-PD-1 efficacy in tumor-bearing mice by increasing gut microbial diversity and regulating microbial composition [33]. A large number of studies showed that polysaccharides from natural resources possessed a lot of efficacy [20,21], including immunoregulatory, anti-tumor, anti-virus, antioxidation, and hypoglycemic activity. It was discovered that chitosan showed antibacterial activities against *E. coli*, which was influenced by its molecular weight, degree of deacetylation, concentration, and pH of the medium. Although this trend was not reflected in this study, it is speculated that this phenomenon was caused by various disease models of mice used in the experiment, accompanied by specific changes in intestinal flora characteristics. Our previous study found that some harmful effects and damage to the colon tissue of the mice were caused by the three-week interference of high-dose (400 mg/kg) EU, and the low-dose intervention group (150 mg/kg) did not produce significantly negative effects compared with the high-dose group. Therefore, the low-dose EU intervention was used in this study to explore whether it would affect the BBR treatment regimen in mice with UC. It was discussed that HPMC could improve the lipid metabolism under fat diet conditions [27]. Based on this fact, we suspected that less energy supply was given in the HPMC group, collectively contributing to the unfavorable result.

According to the above results, the combination of PT, DHP, and BBR is the best choice for the treatment of UC in mice compared with the other three materials. Furthermore, the treatment of combined active compound, BBR, with these two materials shows relatively better efficacy than pure drug treatment. According to the current research, PT used in this experiment plays a vital role in regulating immunity, repairing intestinal barrier function, and improving the diversity of the gut microbiota. An interesting phenomenon was observed in the animal experiment that the mice in the BP group showed significantly better mental states and higher survival rates, although the BP group was also accompanied by weight loss, sticky stools, and bloody stools. It is speculated that this may be contributed to a complex combination of several factors. First, the body’s immune level by inhibiting pro-inflammatory cytokines such as IL-1β was decreased, then the expression of related mRNA was upregulated to increase the amount of tight junction ZO-1 and occludin protein in the colon tissues. At the same time, once the intestinal barrier becomes integrity, it can be in effective control of some gut-derived harmful small molecules such as lipopolysaccharide, reducing the possibility of entering the body’s circulation, which ensures that the systemic inflammatory is downregulated. In addition, the α-diversity of mice in the BP group is significantly better than the DSS group and the BD group. We guess that the increase of the relative abundance of beneficial bacteria can bring more production of small beneficial metabolic such as SCFAs, exerting a comprehensive beneficial effect through the gut–organ axis. It is reasonable to speculate that the more active mental state mentioned above may be closely related to the gut–brain axis by nerves, body fluids, and other pathways.

## 4. Materials and Methods

### 4.1. Chemical and Reagents

BBR was purchased from Nanjing Zelang Biological Technology Co., Ltd. (Nanjing, China). HPMC and EU were purchased from Colorcon (Shanghai, China). CT was purchased from Sigma-Aldrich (Shanghai, China). PT was purchased from Macklin Biochemical Technology Co., Ltd. (Shanghai, China). DHP was purchased from Shanghai Ronghe Pharmaceutical Technology Development Co., Ltd. (Shanghai, China). DSS (MW: 36,000–50,000) was purchased from MP Biomedicals (Santa Ana, CA, USA). The TNF-α, IL-1β, and IL-6 ELISA kits were purchased from Thermo (Waltham, MA, USA). The antibodies, ZO-1, occludin, and claudin-2, were purchased from Proteintech (Rosemont, IL, USA). Radio Immunoprecipitation Assay (RIPA) lysis buffer was purchased from Shanghai Beyotime Co., Ltd. (Shanghai, China). TRIzol Reagent and SYBR Green PCR Master Mix were purchased from Takara (Kusatsu, Shiga, Japan).

### 4.2. Experimental Animals

Six-week-old male C57BL/6 mice (18–20 g) were purchased from Weitonglihua (Beijing, China), they were placed 5 per cage of specific pathogen-free (SPF) conditions under a 12-h light/dark cycle, with an environmental temperature of 23 ± 2 °C, and humidity of 55 ± 5%. All protocols were performed upon approval by the Laboratory Animal Ethics Committee of the Institute of Medicinal Plant Development, Peking Union Medical College (No. SLXD-20210129), and conformed to the Guide for the Care and Use of Laboratory Animals. We followed the ARRIVE guidelines 2.0 regarding animal studies [34].

### 4.3. Induction and Assessment of DSS Colitis

After the one-week adaption phase, animals were randomly distributed into thirteen groups. The first group remained intake of distilled water, other twelve groups were given DSS solution for five consecutive days, they get free access to 5% (*w*/*v* in distilled water) DSS solution in the first two days, then the dose of DSS solution was altered to 3%. At the end of the fifth day, mice obtained normal drinking water for one day. During this period, body weight change, stool consistency, and fecal blood of mice were observed every day for the measurement of DAI. The scores [21] were recorded as follows: body weight (≤1% = 0, 1–5% = 1, 5–10% = 2, 10–15% = 3, ≥15% = 4), stool consistency (normal = 0, loose stool = 2, liquid stool = 4), fecal blood (normal = 0, occult blood = 2, obvious blood = 4).

### 4.4. Drug Intervention

After the induction of DSS colitis, mice were assigned into the following groups: (i) control group, which was given only distilled water; (ii) DSS group treated with distilled water (10 mL/kg); (iii) BBR group that received BBR solution (in distilled water, 100 mg/kg); (iv) drug additives groups (5 groups in total) that were given drug additives solution or suspension (in distilled water, 100 mg/kg), including HPMC, EU, CT, PT, and DHP; (v) combinational groups of BBR and drug additives were intervened by the physical mixture (BH, BE, BC, BP, BD, 100 mg/kg and 100 mg/kg) with the same concentration. Drug intervention experimentation of all groups was completed by oral administration from day 6 to day 12.

### 4.5. Histological Analysis

The distal portion of colon tissues was excised and rinsed with saline, fixed in 4% tissue and cell fixation fluid for 24 h, colon tissues were dehydrated with gradient ethanol (95% for two minutes, 80% for two minutes, 70% for two minutes) and embedded in paraffin wax, then cut into 5μm slice and stained with hematoxylin-eosin (H&E) solution to analyze the histopathological changes in colon tissues [35], and the H&E score was calculated.

### 4.6. Immunohistochemistry Analysis

Immunohistochemistry for ZO-1, occludin, and claudin-2 was performed on a 5 μm slice from the distal colon tissue. Then, the slice was incubated with primary antibody overnight and followed by the biotinylated secondary antibodies [36,37].

### 4.7. Serum Supernatant Enzyme-Linked Immunosorbent Assay (ELISA) Analysis

Mice blood was collected for the detection of the levels of TNF-α, IL-1β, and IL-6 by ELISA kits (Thermo, Waltham, MA, USA). Firstly, adding capture antibody in Corning “Costar” 9018 plates and incubating overnight at 4 °C. Block wells with ELISA diluent were incubated at room temperature for one hour. Next, the samples were transferred to the wells and incubated overnight at 4 °C. The detection antibody and streptavidin-HRP were added to the plate in turn with a different incubation time of one hour and thirty minutes. TMB Solution was added to the wells with incubation for 15 min and the reaction was ended by stop solution [38,39]. Finally, the concentration of cytokine TNF-α, IL-1β, and IL-6 was calculated according to the values of absorbance at 450 nm.

### 4.8. RNA Extraction and RT-qPCR

The colon tissues were quickly frozen and stored at −80 °C. Total RNA of the colon tissues was extracted by using TRIzol reagent, and the concentration of total RNA was detected by Nanodrop 2000 c (Thermo, Waltham, MA, USA). cDNA was synthesized from RNA by SYBR Green PCR Master Mix. RT-qPCR was performed on a LightCycler 960 Instrument (Roche), and GAPDH was selected as the endogenous control for RT-qPCR [40]. The CT value was evaluated for the target transcripts, and the relative mRNA expression of ZO-1, occludin, claudin-2 genes was calculated by the 2^−ΔΔCT^ method.

### 4.9. Microbiome Analysis by 16S rRNA Gene Sequencing

Feces of all mice were collected for further analysis and stored at −80 °C, Microbial community genomic DNA was extracted by utilizing the E.Z.N.A^®^ soil DNA Kit (Omega Bio-Tek, Norcross, GA, USA). The hypervariable region V3–V4 of the bacterial 16S rRNA genes was amplified by an ABI GeneAmp^®^ 9700 PCR thermocycler (ABI, CA, USA). Purified amplicons were pooled in equimolar and paired-end sequenced on an Illumina MiSeqPE300 platform/NovaSeqPE250 platform (Illumina, San Diego, CA, USA) according to the standard protocols by Beijing Expand biotech Science & Technology Co.Ltd. (Beijing, China). The taxonomy of each OTU representative sequence was analyzed by RDP Classifier (version 2.2) against the 16S rRNA database (egg. Silva v138) using a confidence threshold of 0.7.

### 4.10. Statistical Analysis

Results are expressed as the mean ± standard deviation and performed by Origin software (version 2019) and R (version 3.5.1). n = 4–6. Unpaired two-tailed Student’s *t*-test was used to examine significant differences (*p* < 0.05). The significant levels were indicated as follows: * *p* < 0.05, ** *p* < 0.01, *** *p* < 0.001, **** *p* < 0.0001, # *p* < 0.05, ## *p* < 0.01, ### *p* < 0.001, #### *p* < 0.0001, & *p* < 0.05, && *p* < 0.01, &&& *p* < 0.001, &&&& *p* < 0.0001.

## 5. Conclusions

In conclusion, the combined intervention of BBR with PT and DHP shows an excellent beneficial effect on curing UC compared with the supplement of HPMC, EU, and CT, which also exceeds the effect brought by the single intervention of BBR. Our research suggests that PT and DHP may take effect by regulating the relative abundance of Firmicutes, Proteobacteria, and Proteobacteria with polysaccharide fermentation and mucosa-protecting function, contributing to the repair of the intestinal barrier by upregulating the expression of ZO-1 and occludin as well as the decline of the serum TNF-α, IL-1β, and IL-6 level. Furthermore, the mass ratio of BBR to PT of 2:1 has a better repairing effect on the gut microbial imbalance than 1:1 in colitis mice. Therefore, more attention should be paid to the potential effect of pharmaceutical excipients on the active substances to alleviate diseases via regulating gut microbiota.

## Data Availability

The datasets used and analyzed in this study are available from the corresponding author upon request.

## Abbreviations

BBR, berberine; CT, chitosan; DHP, rheum officinale poly-saccharide; DSS, dextran sulphate sodium; DAI, disease activity index; EU, Eudragit S100; ELISA, enzyme-linked immunosorbent assay; HPMC, hydroxypropyl methylcellulose; H&E, hematoxylin-eosin; IBD, inflammatory bowel disease; IHC, Immunohistochemistry; IL, interleukin; JAK, Janus kinase; LPS, lipopolysaccharide; Oncostatin M, OSM; Oncostatin M receptor, OMSR; PT, pectin; SCFAs, short-chain fatty acids; SPF, specific pathogen-free; STAT, signal transducer and activator of transcription; Th17, T helper cell 17; TNF-α, tumor necrosis factor-α; Treg, regulatory T cell; UC, ulcerative colitis; ZO-1, zonula occludens-1.
